# MexAB-OprM Efflux Pump of *Pseudomonas aeruginosa* Offers Resistance to Carvacrol: A Herbal Antimicrobial Agent

**DOI:** 10.3389/fmicb.2019.02664

**Published:** 2019-11-19

**Authors:** Prasanna Vadhana Pesingi, Bhoj Raj Singh, Pavan Kumar Pesingi, Monika Bhardwaj, Shiv Varan Singh, Manoj Kumawat, Dharmendra Kumar Sinha, Ravi Kumar Gandham

**Affiliations:** ^1^Bacteriology & Mycology Division, Indian Veterinary Research Institute, Bareilly, India; ^2^Division of Epidemiology, Indian Veterinary Research Institute, Bareilly, India; ^3^Veterinary Public Health Division, Indian Veterinary Research Institute, Bareilly, India; ^4^Division of Biochemistry, Indian Veterinary Research Institute, Bareilly, India; ^5^Division of Animal Biotechnology, Indian Veterinary Research Institute, Bareilly, India

**Keywords:** mexA, Pseudomonas, efflux, carvacrol, MIC

## Abstract

Carvacrol is a herbal antimicrobial agent with *in vitro* activity against several bacterial pathogens. However, multidrug resistant strains of *Pseudomonas aeruginosa* are resistant to herbal antimicrobial compounds including carvacrol. Resistance of *P. aeruginosa* to carvacrol is not well studied. This study was aimed to identify the gene(s) associated with carvacrol resistance, thus to understand its mechanisms in *P. aeruginosa*. A herbal drug resistant strain was isolated from a hospital environment. Carvacrol sensitive mutant was generated using transposon mutagenesis. The inactivated gene in the mutant was identified as *mexA*, which is part of the *mexAB-oprM* operon. Inactivation of the *mexA* gene resulted in a >31-fold reduction in MIC of carvacrol, whereas a >80-fold reduction was observed in the presence of drug efflux inhibitor phenylalanine-arginine β-naphthylamide (PAβN). The parental herbal-resistant strain was completely killed within 3 h of incubation in the presence of carvacrol and PAβN. The *mexA* inactivation did not affect the resistance to other herbal compounds used. The results demonstrate that resistance to carvacrol in *P. aeruginosa* is mediated by the MexAB*-*OprM efflux pump.

## Introduction

*Pseudomonas aeruginosa* is a Gram-negative bacteria present ubiquitously in nature and is one of the major causative agents of the nosocomial infections worldwide (Lederberg, 2000; Quartin et al., 2013). This organism produces a broad range of virulence factors and is associated with a variety of infections (Tang et al., 1996; Lamont et al., 2002). Recently, WHO classified *P. aeruginosa* as one of the critical pathogens in its first published list of antibiotic-resistant priority pathogens based on the urgency of need for new antibiotics (World Health Organization, 2017; Willyard, 2017).

*Pseudomonas aeruginosa* is intrinsically resistant to many antimicrobial agents which can be mediated by restricted uptake of antimicrobials through the outer membrane, by expression of efflux pumps and/or by the action of drug degrading enzymes (Li et al., 2015). The intrinsic resistance through efflux pumps could be achieved by constitutive basal level expression of efflux pumps (Okamoto et al., 2001). The acquired drug resistance can be attained by mutations at chromosomal genes coding for regulatory proteins. The efflux pump systems, MexAB-OprM and MexXY-OprM were well recognized in *P. aeruginosa* (Li et al., 1995; Aires et al., 1999). The MexAB-OprM system is responsible for the resistance to quinolones, macrolides, novobiocin, chloramphenicol, tetracyclines, lincomycin, and β-lactam antibiotics (Li et al., 1995; Masuda et al., 2000).

For the last two decades, the scientific community could not add any new class of antibiotics in spite of immense research. On the other hand, the emergence and spread of multidrug resistant infections and complications arising from antibiotic therapy, has drawn attention on alternative medicines including traditional herbal medicines to identify novel bioactive compounds.

Among herbal preparations, essential oils of several medicinal plants are often shown to possess antimicrobial properties. The essential oil of cinnamon has been found the most effective, followed by the essential oil of oregano and thyme (Aggarwal et al., 2000). Some essential oils have proven to kill biofilms of *P. aeruginosa*, *Pseudomonas putida*, and *Staphylococcus aureus* (Kavanaugh and Ribbeck, 2012).

Carvacrol is one of the active ingredients in thyme and oregano oils and exerts a broad spectrum of antimicrobial activity against both Gram-positive and Gram-negative bacteria. It exerts bacteriostatic and bactericidal activities against *Campylobacter jejuni*, *Listeria monocytogenes*, *S. aureus*, *Staphylococcus epidermidis*, *Lactobacillus sakei*, *P. putida*, *Streptococcus mutans*, and *Bacillus subtilis* (Lambert et al., 2001; Friedman et al., 2002; Suntres et al., 2015).

However, the microbes are known to adapt to different antimicrobial substances in their environments. The rise of such herbal drug resistant microbial strains have been reported in the past but the detailed study of molecular mechanism of this resistance to many herbal compounds are yet to be explored.

In the current study, we have revealed the mechanism of carvacrol resistance. Initially, we have isolated carvacrol resistant *P. aeruginosa* from environmental sources. Using random transposon mutagenesis and next generation sequencing approaches, we have identified carvacrol sensitive mutant that carried the inactivated *mexA* gene. The role of MexAB-OprM in carvacrol resistance was assessed by time-killing assay in the presence of an efflux pump inhibitor (EPI).

## Materials and Methods

### Bacterial Strains, Culture Condition, and Herbal Antimicrobials

Carvacrol resistant *P. aeruginosa* strain PA-Y7 was isolated from the hospital environmental samples Pondicherry, India using 4% carvacrol strips (prepared in our laboratory) (Supplementary Figure S1) and confirmed by biochemical tests such as methyl red, voges proskauer, nitrate reduction, malonate utilization, Tween 20 hydrolysis, and gelatin hydrolysis (Singh, 2009) and PCR (Spilker et al., 2004) (Supplementary Figure S2). The strain PA-Δ*mexA* is a *mexA* mutant of PA-Y7. The organisms were cultured in suitable media and incubated at 37°C for overnight. The media was supplemented with kanamycin (50 μg/mL) or varying concentrations of carvacrol whenever required. The details of the primers used in the study were indicated in Table 1.

**TABLE 1:** Primers used in this study.

**Purpose**	**Sequence (5′ → 3′)**	**References**
*Pseudomonas*	Forward: GACGGGTGAGTAATGCCTA	Spilker et al., 2004
genus specific	Reverse: CACTGGTGTTCCTTCCTATA	
*P. aeruginosa*	Forward: GGGGGATCTTCGGACCTCA	Spilker et al., 2004
species specific	Reverse: TCCTTAGAGTGCCCACCCG	
EZ-Tn5^TM^	Forward: AATCAGGTGCGACAATCTATC	This study
≪ > *KAN*−2 ≫Tnp	Reverse: GAAATCACCATGAGTGACGAC	
transposon		

### Generation of Carvacrol Sensitive Mutant

The carvacrol sensitive mutants were generated by random mutagenesis using EZ-Tn5^TM^<*KAN*−2> ≫Tnp^TM^ Transposome kit (Epicenter, United States). Electrocompetent cells of *P. aeruginosa* were prepared according to the protocol described by Dawoud et al. (2014) with slight modifications. Briefly, a single colony was inoculated in 10 ml of trypticase soy broth (TSB) (BD, United States) and incubated at 37°C, 180 rpm for overnight. Sub-culturing was done at 1: 100 in 100 ml TSB at 37°C, 180 rpm until OD_600_ reaches 0.6. The culture was centrifuged for 10 min at 7000 × *g* at 4°C. The bacterial pellet was washed sequentially in 25, 15, 10, 5, 2, and 1 ml sterile ice-cold 10% glycerol. Finally, the pellet was suspended in 500 μl of 10% glycerol and kept on ice. The cells were electroporated at 2.5 kV for 5 msec in a multiporator (Eppendorf). The cells were transferred to 5 ml of fresh Luria Bertani (LB) broth (BD, United States) and incubated at 37°C at 180 rpm for 2 h. The cell suspension was spread on trypticase soy agar (TSA) (BD, United States) containing 50 μg/mL of kanamycin (kanamycin resistance is the resistance marker of Transposon). Agar plates were allowed to dry and incubated at 37°C for overnight. Approximately, 1300 transposon mutants were analyzed for carvacrol sensitivity. A fresh culture of each mutant was spot inoculated on TSA plate containing carvacrol (2.5 mg/mL) and incubated at 37°C for overnight. The selected carvacrol sensitive mutants were further confirmed for transposon insertion by PCR (Table 1). The whole genomic DNA was extracted from selected mutants as well as parent strain using QIAmp DNA isolation kit (Qiagen, United States). The DNA was concentrated by using the SpeedVac (Eppendorf) and the concentration was measured by nanodrop (Eppendorf). Mapping of transposon insertion was done by next-generation sequencing (NGS) at Bioserve Biotechnology, Hyderabad, India.

### Determination of Herbal Drug Sensitivity in PA-Y7 and PA-Δ*mex*A

PA-Y7 and PA-Δ*mexA* were screened against 14 herbal compounds as per the Kirby Bauer method (Hudzicki, 2009). Carvacrol (antimicrobial component of several essential oils including oregano, thyme, ajowan etc.), cinnamon oil (*Cinnamomum verum*), cinnamaldehyde (active component of cinnamon oil), lemongrass oil (*Cymbopogon citratus*) and citral oil (active component of lemongrass oil) were purchased from Sigma-Aldrich, United States. Agarwood (*Aquilaria malaccensis*) oil, ajowan (*Trachyspermum ammi*) oil, betel (*Piper betle*) leaf oil, guggul (*Commiphora wightii*) oil, holy basil (*Ocimum sanctum*) oil, patchouli (*Pogostemon cablin*) oil, sandalwood (*Santalum album*) oil and *Zanthoxylum rhetsa* seed coat essential oil were procured from the Shubh Flavours and Fragrances Pvt. Ltd., Delhi, India. Guggul oil was received from Dr. Mahtab Z. Siddiqui, Principal Scientist, Processing and Product Development Division, Indian Institute of Natural Resins and Gums (IINRG), India. These tested herbal compounds possess a purity of 99% to 99.9%. Each herbal disc contains 1 mg per microliter of pure herbal compound. The lawn cultures were prepared from freshly grown culture (∼ OD_600_ 0.3–0.6) on Mueller Hinton agar (MHA) plates (BD, United States). The discs containing herbal compounds were placed and the plates were incubated at 37°C for overnight. In addition, the antibiotic discs with chloramphenicol, ciprofloxacin, colistin and polymyxin B were used as control.

### MIC Determination and Efflux Pump Inhibition Assay

The MIC of carvacrol for strains PA-Y7 and PA-Δ*mexA* were analyzed using agar well dilution method on MHA. Different dilutions of carvacrol ranging from 0.01 to 10 mg/mL were loaded into the wells and incubated. The MIC of phenylalanine-arginine β-naphthylamide (PAβN) for PA-Y7 was determined by the broth dilution method as described by the Mawabo et al. (2015) with some modifications. Briefly, 1 mL of PAβN (at 400 μg/mL) was added to a tube containing 1 mL of LB and two fold dilution was done till PAβN concentration reaches 12.5 μg/mL. About 100 μl of freshly grown culture (∼ OD_600_ to 0.6) was added to all the tubes and kept for incubation. The MIC was determined by lowest concentration of PAβN inhibiting the visible growth in tubes. For the determination of efflux pump inhibition, two sets of carvacrol dilutions were used. The final concentrations of first set of dilutions were 10, 9, 8, 7, 6, 5, 4, 3, 2, and 1 mg/mL and the second set of dilutions made to get final concentration of 1, 0.5, 0.25, 0.125, 0.062, and 0.031 mg/mL. One hundred and twenty five microliters of PAβN (i.e., 50 μg/mL) or phosphate buffered saline (PBS) was added to a series of tubes containing 725 μl of LB broth, 50 μl of carvacrol and 100 μl of freshly grown culture (∼ OD_600_ to 0.6). The tubes were incubated at 37°C for overnight.

### Time-Kill Assay

Different combinations carvacrol and PAβN (PAβN 50 μg/mL + carvacrol 5 mg/mL; PAβN 25 μg/mL + carvacrol 10 mg/mL; PAβN 50 μg/mL + carvacrol 10 mg/mL; PAβN 25 μg/mL + carvacrol 125 μg/mL) were added to tubes. The cells of PA-Y7 were added to these tubes to a final concentration of 1.5 × 10^5^ CFU/mL. Aliquots were drawn at 0, 3, 6, 12, and 24 h incubation and serially diluted in PBS. The dilutions were plated on TSA and incubated at 37°C for overnight. Culture control, carvacrol control and PAβN control were maintained.

## Results

### Mapping of Transposon Site in Carvacrol Sensitive Mutant

**FIGURE 1:**
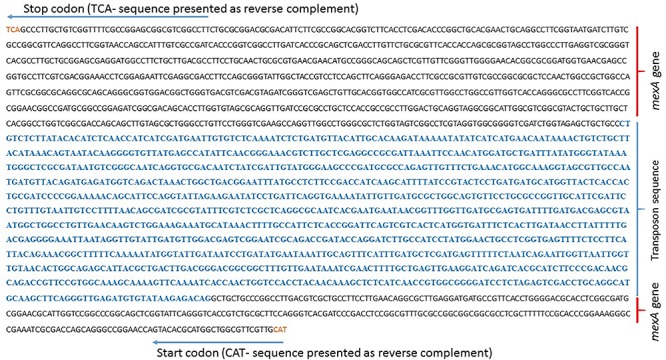
Location of 1221 bp of transposon sequence in the genome of *P. aeruginosa* mutant PA-Δ*mexA.* The raw data from NGS was retrieved and made into contigs using CLC genomics workbench. Local BLAST was performed to locate transposon sequence in contigs and 1221 bp of transposon sequence in contigs was located by MacVector software.

Initially, we have confirmed the transposon insertion by resistance to kanamycin as well as by amplifying a segment of transposon in mutant (Supplementary Figure S3). Further, by using MacVector software, the insertion of 1221 bp transposon sequence was located from the NGS data of carvacrol sensitive mutant (Figure 1). About 200 nucleotides each from upstream and downstream of the transposon insertion site were NBLAST in NCBI database as well as Pseudomonas genome database^1^ (Winsor et al., 2016) by taking *P. aeruginosa* PAO1 as a reference strain. The sequence matched with the sequence of the *mexA* gene of *P. aeruginosa* PAO1 strain with 100% identity (Supplementary Figure S4) suggesting that *mexA* gene was inactivated by the insertion of the transposon.

### Herbal Drug Sensitivity in PA-Y7 and PA-Δ*mex*A

The PA-Y7 strain showed resistance to all the herbal compounds tested whereas the PA-Δ*mexA* has become sensitive to carvacrol, cinnamon oil, thyme oil, and cinnamaldehyde but not to other herbal compounds. The control discs chloramphenicol and ciprofloxacin showed 14 and 24 mm in PA-Y7 and 22 and 32 mm in PA-Δ*mexA*, respectively. The colisin (9 mm in both strains) and polymyxin B (10 mm in PA-Y7 and 11 mm in PA-Δ*mexA*) did not show difference in zone of inhibition between these strains (Supplementary Figure S5).

### Efflux Pump Inhibition Reduced Carvacrol MIC in the Parent Strain

**FIGURE 2:**
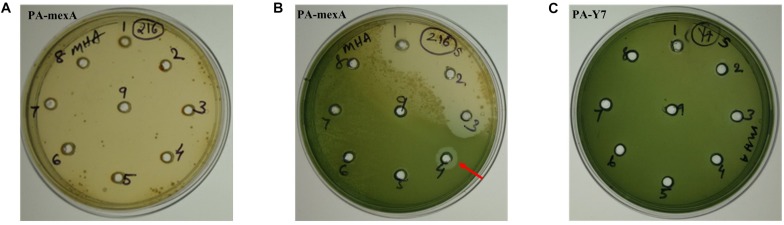
Determination of MIC of carvacrol for PA-mexA and PA-Y7 by agar well method. Lawn culture was made on MHA plates containing wells and different concentrations of carvacrol **(A,C)** well number 1–9 contains 2, 3, 4, 5, 6, 7, 8, 9, and 10 mg/mL, respectively; **(B)** well number 1–9 contains 0.01, 0.02, 0.04, 0.08, 0.16, 0.32, 0.64, 1.28, and 2.56 mg/mL, respectively, were added. The minimum concentration of carvacrol that inhibited growth of mutant was 320 μg/mL (indicated by arrow). **(C)** PA-Y7 was resistant to carvacrol at 10,000 μg/mL.

**FIGURE 3:**
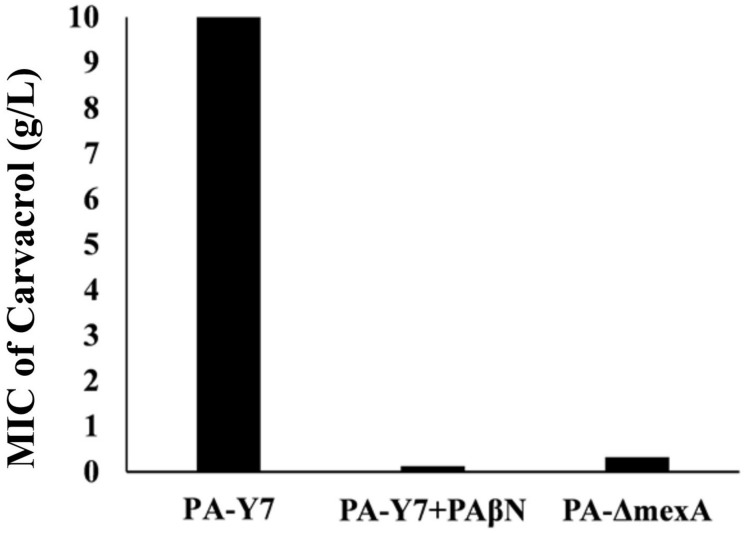
Comparison of MIC values of carvacrol for wild type and *mexA* mutant strains. The MIC value for wild type (PA-Y7) was found to be more than 10 mg/mL, whereas the MIC for PA-Y7 reduced to 0.125 mg/mL when treated with efflux pump inhibitor PAβN. The mutant having inactivated *mexA* gene (PA-Δ*mexA*) has carvacrol MIC of 0.32 mg/mL.

The MIC values of carvacrol and PAβN for parent strain were estimated to be >10 mg/mL and >0.2 mg/mL, respectively. The MIC of carvacrol for PA-Δ*mexA* mutant was significantly reduced to at least 31.25 folds (0.32 mg/mL) (Figure 2). Further, inhibition of efflux pumps by PAβN significantly reduced carvacrol MIC to at least 80 folds (0.125 mg/mL) in parent strain (Figure 3). This suggests that carvacrol or its metabolite should be a substrate of MexAB-OprM efflux pump.

### Time-Kill Assay

**FIGURE 4:**
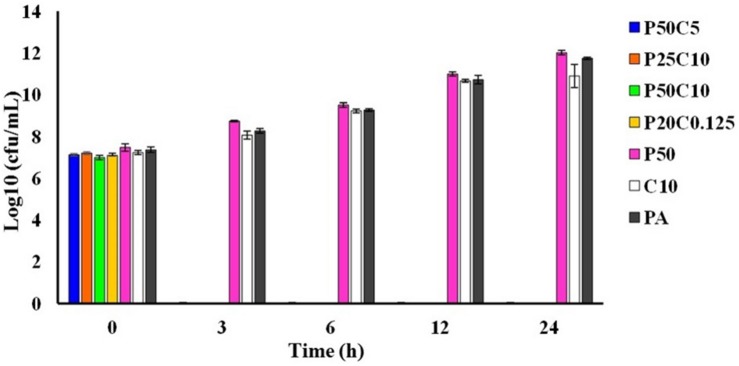
Time-killing assay of carvacrol and PAβN combinations against *P. aeruginosa* PA-Y7. Different combinations carvacrol and PAβN-P50C5 (PAβN 50 μg/mL + carvacrol 5 mg/mL; P25C10 (PAβN 25 μg/mL + carvacrol 10 mg/mL); P50C10 (PAβN 50 μg/mL + carvacrol 10 mg/mL); P25C0.125 (PAβN 25 μg/mL + carvacrol 125 μg/mL) were added to tubes containing 1.5 × 10^5^ CFU/mL of PA-Y7. Aliquots were drawn at different intervals, serially diluted in PBS and plated on TSA plates. The CFU/mL was determined.

Time-kill assay has been performed by mixing 10 or 5 or 0.125 mg/mL of carvacrol to 0.025 or 0.05 mg/mL of PAβN (named as P50C5, P25C10, P50C10, and P25C0.125). Figure 4, showing the results of the time kill assay where, *P. aeruginosa* was completely killed in 3 h of incubation in all combinations of PAβN and carvacrol whereas, in controls (named as P50, C10, and PA) were shown pronounced cell growth till 24 h.

## Discussion

*Pseudomonas aeruginosa* well known for multidrug resistance is responsible for 10% hospital-borne infections worldwide (Morrison and Wenzel, 1984; Aloush et al., 2006). *P. aeruginosa* has not only emerged as MDR pathogen but also evolved as extensively drug resistant (XDR) and pan drug resistant (PDR) strain as well (Gill et al., 2016). Evolution of drug resistance has led an attention on the traditional medicines such as herbal drugs. The latter have been used for the treatment of many infectious diseases in humans as well as animals all over the world (Verma and Singh, 2008). Some essential oils have been reported to kill biofilms formed by *P. aeruginosa* (PAO1), *P. putida*, and *S. aureus* (Kavanaugh and Ribbeck, 2012).

Carvacrol [2-methyl-5-(methyl ethyl) phenol] is one of the major components of oregano oil which is known for its wide spectrum of antimicrobial activity *in vitro* (De Martino et al., 2009). Carvacrol possesses several biological properties which include anti-inflammatory, antioxidant, anti-leishmanial, hepatoprotective, antimicrobial, antitussive, antispasmodic, and antitumoral activities (Robledo et al., 2005). Several studies have been conducted and proved that carvacrol has both bacteriostatic and bactericidal activity against microorganisms such as *Escherichia coli* (Friedman et al., 2002; Juneja and Friedman, 2007; Perez-Conesa et al., 2011), *Clostridium perfringens* (Juneja and Friedman, 2007), *Pseudomonas fluorescens* (Lambert et al., 2001; Ben Arfa et al., 2006), *Lactobacillus plantarum* (Ben Arfa et al., 2006*), Saccharomyces cerevisiae* (Ben Arfa et al., 2006), *Botrytis cinerea* (Ben Arfa et al., 2006), *Shigella* spp. (Bagamboula et al., 2004). It is also proved to be effective against methicillin-resistant strains of *S. aureus* and *S. epidermidis* (Nostro et al., 2009). Encapsulated carvacrol of surfactant micelles proven effective in inhibiting the growth of *E. coli* O157:H7 and *L. monocytogenes* (Perez-Conesa et al., 2011).

However, many microorganisms are resistant to herbal compounds. For instance, Khan et al. (2009) reported that *P. aeruginosa*, *E. coli*, *Klebsiella pneumoniae* and *Candida albicans* were resistant to many herbal antimicrobials. Similarly, *E. coli*, *P. aeruginosa*, and *Shigella flexneri* were resistant to aqueous extracts of unripe banana (*Musa sapientum*), lemongrass (*Cymbopogon citratus*) and turmeric (*Curcuma longa*) (Fagbemi et al., 2009). Sage essential oil was found to be ineffective against *S. aureus*, *B. subtilis*, *P*. *aeruginosa*, *Salmonella typhimurium* (Bosnić et al., 2006). The reports on the ineffectiveness of herbal drugs among certain bacterial strains and their herbal antimicrobial compounds resistance cannot be neglected (Vadhana et al., 2015). The mechanisms of microorganisms to resist herbal antimicrobial compounds are not well studied. This study has been carried out to understand the mechanism of carvacrol resistance in *P*. *aeruginosa.*

It was reported that in *P. aeruginosa*, resistance to antibiotics and some flavonoids are mediated through various efflux pumps (Papadopoulos, 2008). Here, we have created a carvacrol sensitive mutant of *P. aeruginosa* from a carvacrol resistant strain by transposon insertional inactivation. The inactivated gene in the mutant was found to be *mexA*, which is part of the *mexAB-oprM* operon. The *mexAB-oprM* operon encodes the MexAB-OprM efflux system, a member of resistance-nodulation-cell division (RND) family of exporters with broad substrate specificity (Morita et al., 2012; Li et al., 2015). The protein MexA is lipoprotein in nature but it can function without a lipid moiety as well (Yoneyama et al., 2000). The function of MexA protein is to link MexB to the outer membrane porin-like OprM, thereby facilitating one-step efflux of drugs out of the cell (Ma et al., 1994). MexB functions in the proton motive force driven efflux of antibiotics across the cytoplasmic membrane (Poole et al., 1996). MexAB-OprM efflux pump is responsible for resistance to various classes of antibiotics such as β-lactams, β-lactam inhibitors, fluoroquinolones, tetracyclines, tigecycline, novobiocin, thiolactomycin, sulfonamides, macrolides, aminoglycosides, etc. (Poole, 2011; Li et al., 2015). In this study, PA-Δ*mexA* as compared to PA-Y7 strain has showed significant increase in the zone of inhibition of chloramphenicol and ciprofloxacin which are the substrates of the MexAB-OprM pump but not for colistin and polymyxin B antibiotics. Resistance to carvacrol is likely attributable to the MexAB-OprM efflux pump. The relationship between carvacrol and RND systems has been previously demonstrated in other bacterial species (Cirino et al., 2014). Further, *Thymus maroccanus* essential oil and its major components (carvacrol and thymol) were able to select variants of *E. coli* that overexpress the AcrAB efflux pump (Fadli et al., 2014). Carvacrol and thymol enhanced accumulation of ethidium bromide in various pathogens including *P. aeruginosa* (Miladi et al., 2016). To date, there are no reports on the role of *mex*A gene or *mex*AB*-opr*M operon on carvacrol resistance but their role in tea tree essential oil tolerance in *P. aeruginosa* has been reported (Papadopoulos, 2008).

The MIC of carvacrol in PA-Δ*mexA* mutant was significantly reduced. This indicates that MexAB-OprM efflux pump is likely responsible for carvacrol resistance. The role of efflux pumps has been well studied using EPIs. In the current study, we have used PAβN, a well studied EPI in *P. aeruginosa* (Lomovskaya et al., 1999). PAβN effectively reduced the MIC of drugs such as levofloxacin, chloramphenicol, carbenicillin, erythromycin which are substrates of MexAB-OprM pump (Opperman and Nguyen, 2015). Here, the MIC of PAβN was >0.2 mg/mL, however, we have used 0.05 mg/mL of PAβN due to its membrane damaging effect at higher concentrations. The MIC of carvacrol was significantly reduced by >80 folds in the presence of PAβN. Together the results suggest that carvacrol is the substrate of the MexAB-OprM efflux pump that imparts resistance to carvacrol. The lower MIC of carvacrol was noticed in EPI treatment of the parent strain as compared *mexA* mutant. This may be due to the broad range EPI activity of PAβN against other pumps. Time-kill assay has indicated that the combinations of carvacrol and PAßN killed *P. aeruginosa* within 3 h *in vitro* but failed to do so as individual components, indicating their synergistic action. To our knowledge, this is the first study to report the role of an efflux pump in carvacrol resistance in *P. aeruginosa.* Surprisingly, inactivation of the *mexA* gene did not affect the resistance to other herbal compounds such as essential oils from lemongrass, sandalwood, ajowan, betel leaf, guggal, patchouli, agar, holy basil, citral, and methanolic extracts from zanthoxylum and kalonji.

## Data Availability Statement

All datasets generated for this study are included in the article/Supplementary Material.

## Author Contributions

PVP: main research work and manuscript writing. BS: mentor, research work designing, manuscript writing, and other technical guidance. PKP: assistance in transposon mutagenesis, mutant screening, efflux pump inhibition assay. MB: DNA isolation for NGS and processing samples for NGS. SS: sample collection. MK: assistance mutant screening. DS: biochemical test and technical assistance. RG: co-mentor and NGS data analysis.

## Conflict of Interest

The authors declare that the research was conducted in the absence of any commercial or financial relationships that could be construed as a potential conflict of interest.

## Footnotes

1 http://www.pseudomonas.com
