# Supplementary material for: MexAB-OprM Efflux Pump of Pseudomonas aeruginosa Offers Resistance to Carvacrol: A Herbal Antimicrobial Agent
Source: Front Microbiol. 2019 Nov 19;10:2664. doi: 10.3389/fmicb.2019.02664 (PMC6877666; doi:10.3389/fmicb.2019.02664)
Supplement: Supplementary file 1 [file Presentation_1.PPTX]

## Slide 1
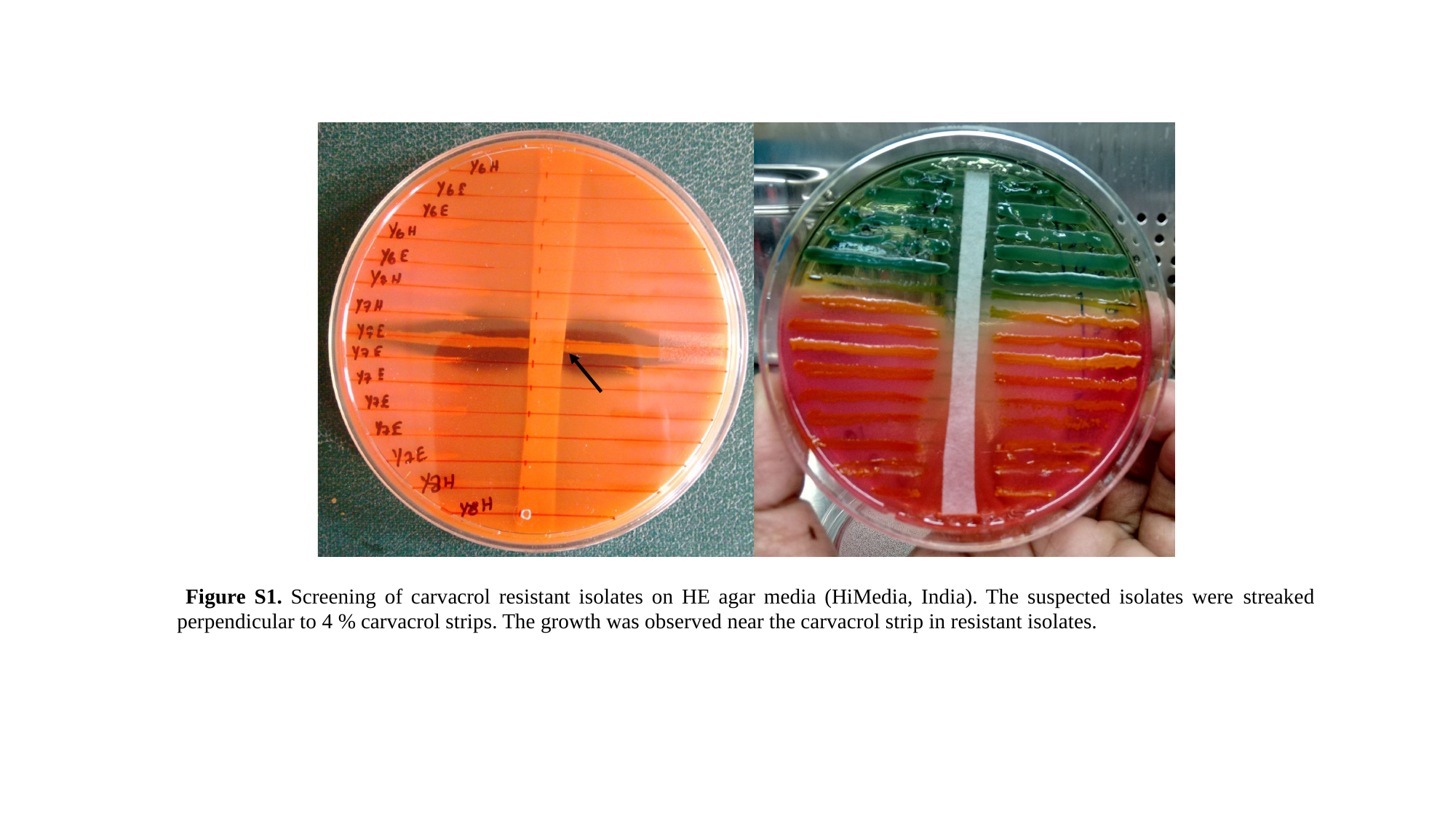

Figure S1. Screening of carvacrol resistant isolates on HE agar media (HiMedia, India). The suspected isolates were streaked perpendicular to 4 % carvacrol strips. The growth was observed near the carvacrol strip in resistant isolates.

## Slide 2
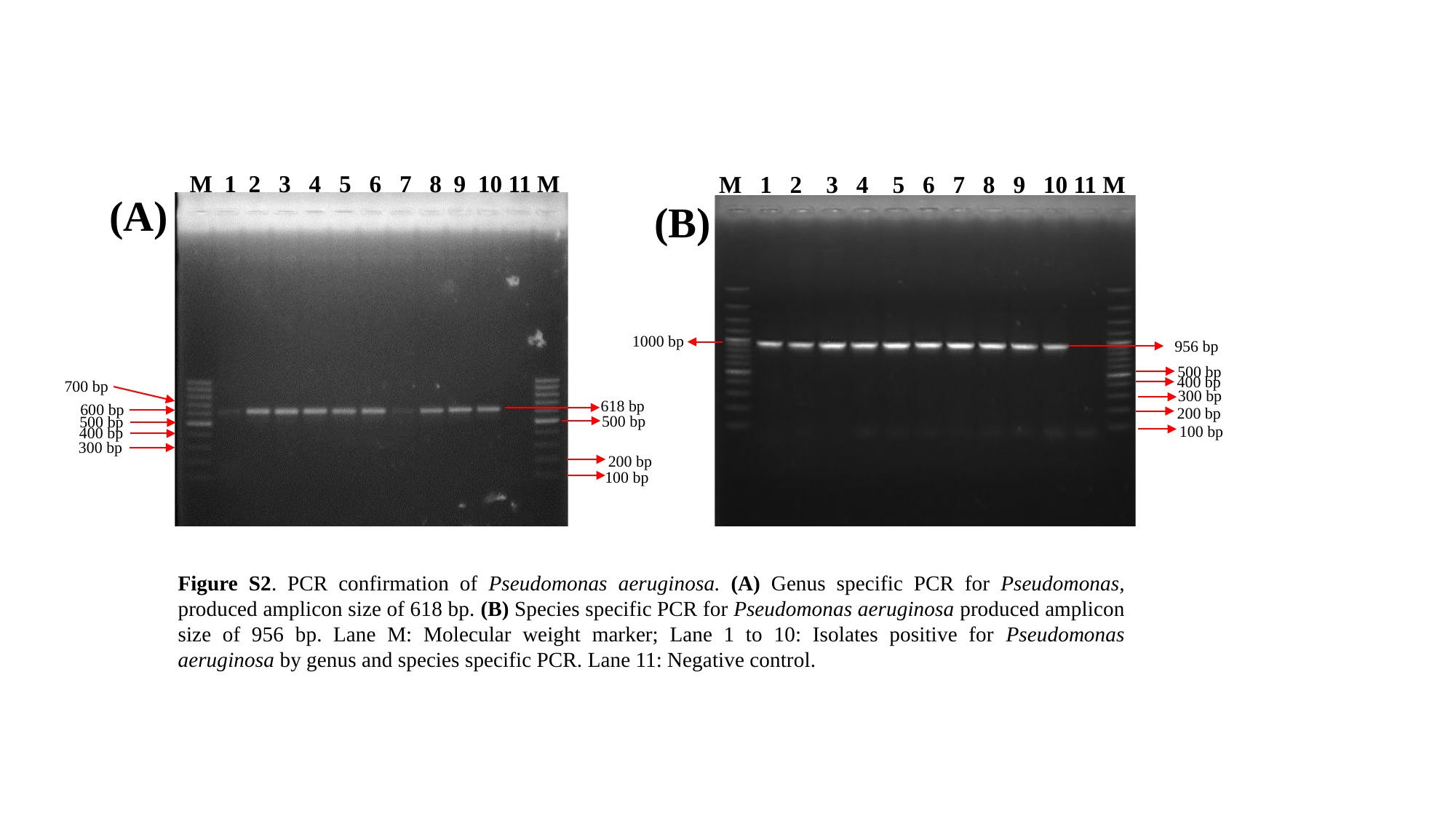

M 1 2 3 4 5 6 7 8 9 10 11 M
M 1 2 3 4 5 6 7 8 9 10 11 M
(A)
(B)
1000 bp
956 bp
500 bp
400 bp
700 bp
300 bp
618 bp
600 bp
500 bp
200 bp
500 bp
500 bp
100 bp
400 bp
300 bp
200 bp
100 bp
Figure S2. PCR confirmation of Pseudomonas aeruginosa. (A) Genus specific PCR for Pseudomonas, produced amplicon size of 618 bp. (B) Species specific PCR for Pseudomonas aeruginosa produced amplicon size of 956 bp. Lane M: Molecular weight marker; Lane 1 to 10: Isolates positive for Pseudomonas aeruginosa by genus and species specific PCR. Lane 11: Negative control.

## Slide 3
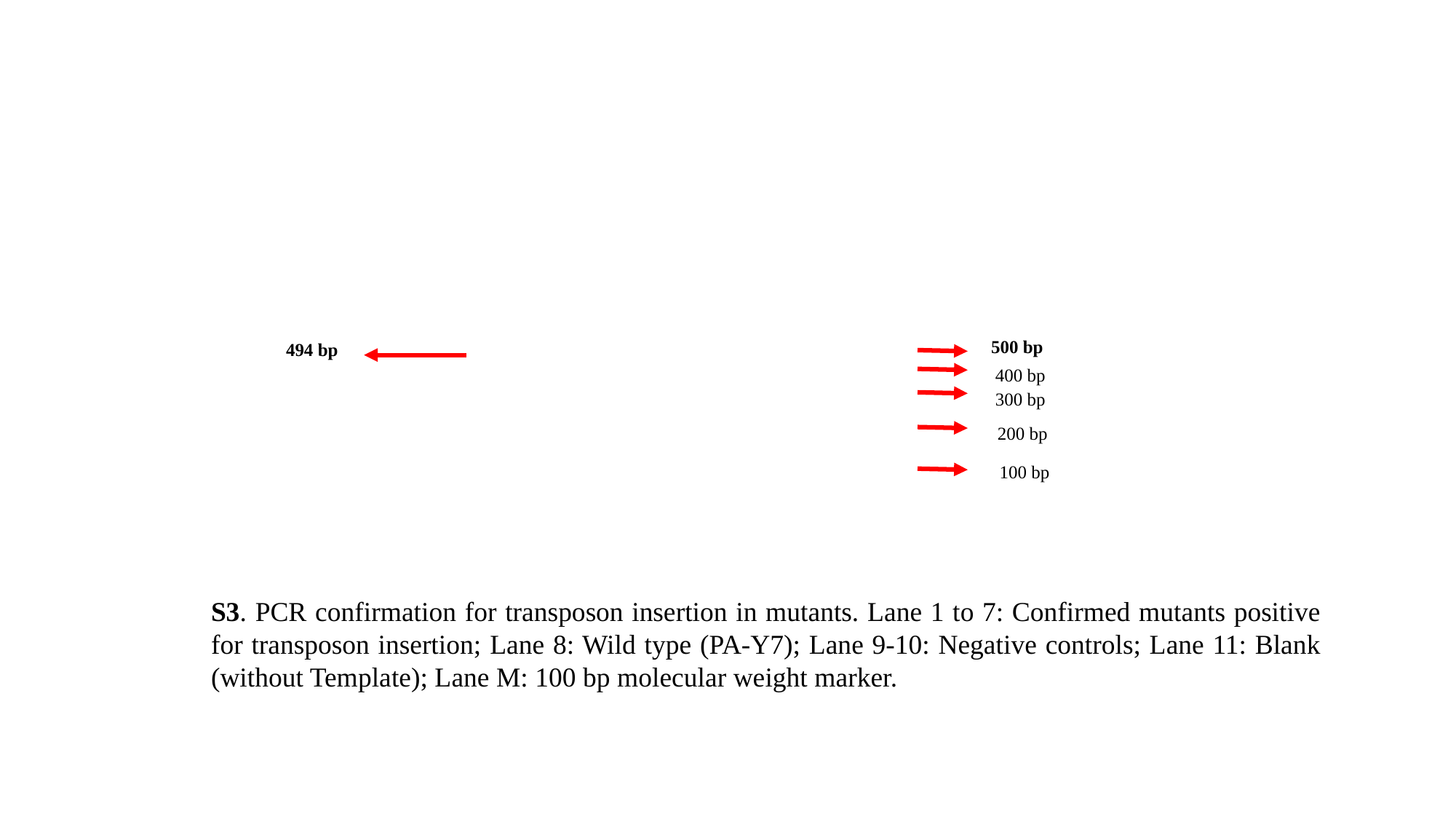

M 1 2 3 4 5 6 7 8 9 10 11 M
500 bp
494 bp
400 bp
300 bp
200 bp
100 bp
S3. PCR confirmation for transposon insertion in mutants. Lane 1 to 7: Confirmed mutants positive for transposon insertion; Lane 8: Wild type (PA-Y7); Lane 9-10: Negative controls; Lane 11: Blank (without Template); Lane M: 100 bp molecular weight marker.

## Slide 4
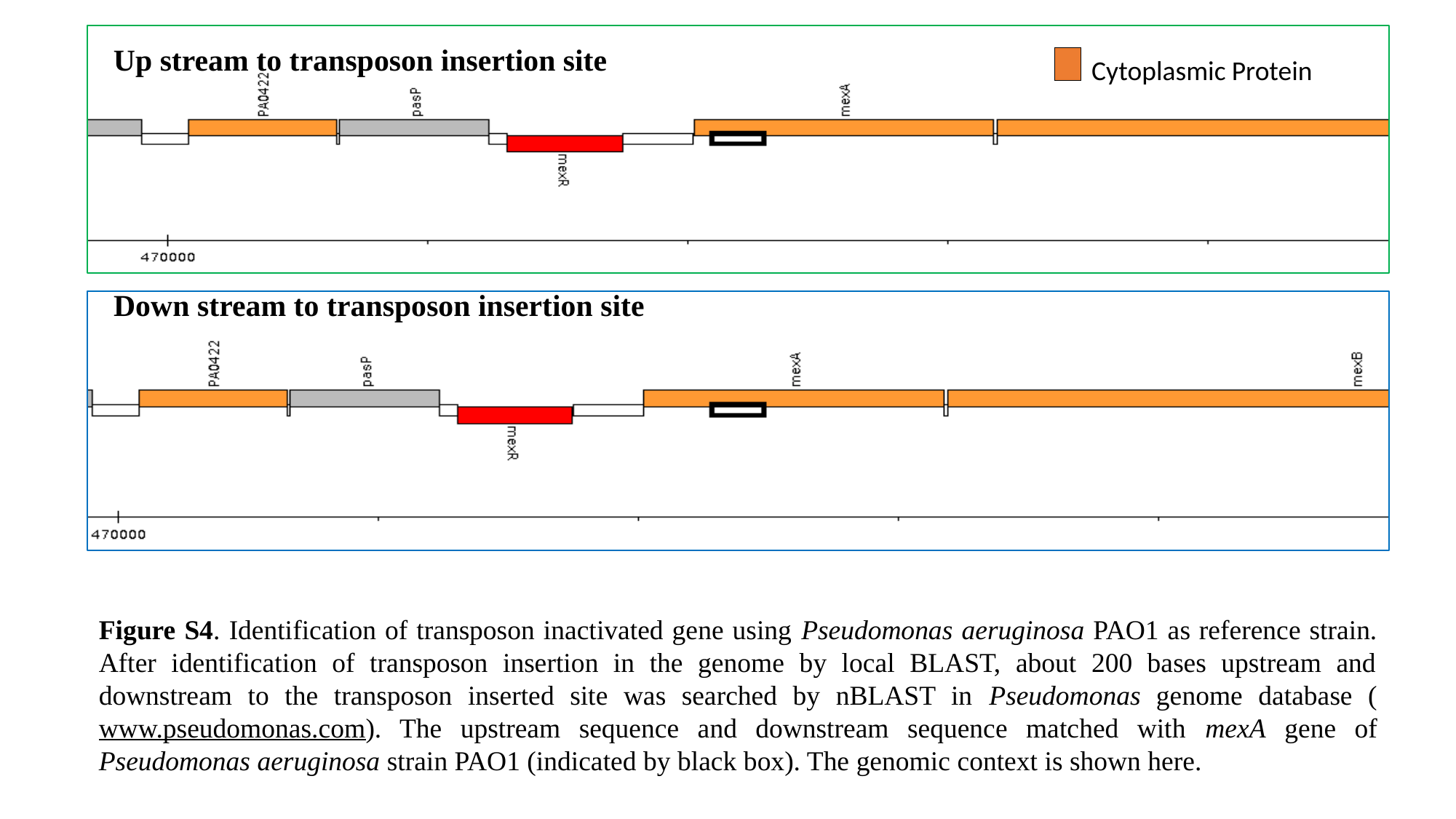

Up stream to transposon insertion site
Cytoplasmic Protein
Down stream to transposon insertion site
Figure S4. Identification of transposon inactivated gene using Pseudomonas aeruginosa PAO1 as reference strain. After identification of transposon insertion in the genome by local BLAST, about 200 bases upstream and downstream to the transposon inserted site was searched by nBLAST in Pseudomonas genome database (www.pseudomonas.com). The upstream sequence and downstream sequence matched with mexA gene of Pseudomonas aeruginosa strain PAO1 (indicated by black box). The genomic context is shown here.

## Slide 5
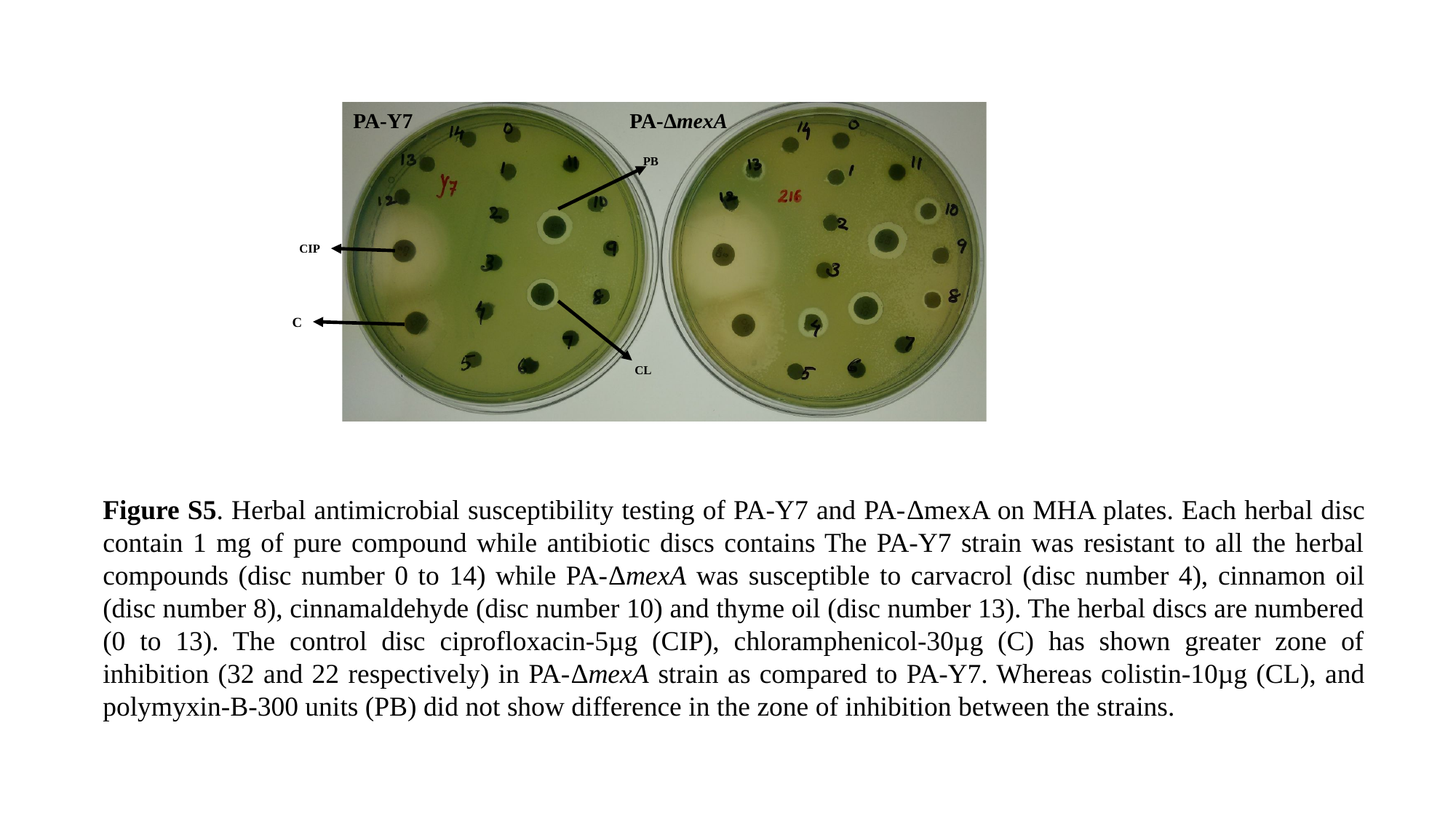

PA-Y7
PA-ΔmexA
PB
CIP
C
CL
Figure S5. Herbal antimicrobial susceptibility testing of PA-Y7 and PA-ΔmexA on MHA plates. Each herbal disc contain 1 mg of pure compound while antibiotic discs contains The PA-Y7 strain was resistant to all the herbal compounds (disc number 0 to 14) while PA-ΔmexA was susceptible to carvacrol (disc number 4), cinnamon oil (disc number 8), cinnamaldehyde (disc number 10) and thyme oil (disc number 13). The herbal discs are numbered (0 to 13). The control disc ciprofloxacin-5µg (CIP), chloramphenicol-30µg (C) has shown greater zone of inhibition (32 and 22 respectively) in PA-ΔmexA strain as compared to PA-Y7. Whereas colistin-10µg (CL), and polymyxin-B-300 units (PB) did not show difference in the zone of inhibition between the strains.
